# Mobile Apps for Blood Pressure Monitoring: Systematic Search in App Stores and Content Analysis

**DOI:** 10.2196/mhealth.9888

**Published:** 2018-11-14

**Authors:** Hussein Jamaladin, Tom H van de Belt, Lianda CH Luijpers, Falco R de Graaff, Sebastian JH Bredie, Nel Roeleveld, Marleen MHJ van Gelder

**Affiliations:** 1 Department for Health Evidence Radboud Institute for Health Sciences Radboud University Medical Center Nijmegen Netherlands; 2 Radboud REshape Innovation Center Radboud University Medical Center Nijmegen Netherlands; 3 Department of Internal Medicine Radboud University Medical Center Nijmegen Netherlands

**Keywords:** mobile phone, mHealth, app review, high blood pressure, self-management, mobile app

## Abstract

**Background:**

Using a mobile app for self-management could make it easier for patients to get insight into their blood pressure patterns. However, little is known about the availability, quality, and features of mobile apps targeting blood pressure.

**Objective:**

The goal of the research was to determine the availability, functionality, and quality of mobile apps that could be used for blood pressure monitoring purposes.

**Methods:**

A systematic app search was performed based on the standards for systematic reviews. We searched the Dutch official app stores for Android and iOS platforms using predefined keywords and included all English and Dutch mobile apps targeting blood pressure. Two independent assessors determined eligibility and quality of the apps using the 5-point Mobile App Rating Scale (MARS). Quality scores of the apps with and without 17 a priori selected characteristics were compared using independent sample t tests.

**Results:**

A total of 184 apps (104 Android, 80 iOS) met the inclusion criteria. The mean overall MARS score was 2.63 (95% CI 2.55-2.71) for Android and 2.64 (95% CI 2.56-2.71) for iOS. The apps Bloeddruk (4.1) and AMICOMED BP (3.6) had the highest quality scores on the Android and iOS platforms, respectively. Of the app characteristics recorded, only pricing, in-app advertisements, and local data storage were not associated with the quality scores. In only 3.8% (7/184) of the apps, involvement of medical experts in its development was mentioned, whereas none of the apps was formally evaluated with results published in a peer-reviewed journal.

**Conclusions:**

This study provides an overview of the best apps currently available in the app stores and important key features for self-management that can be used by health care providers and patients with hypertension to identify a suitable app targeting blood pressure monitoring. However, the majority of the apps targeting blood pressure monitoring were of poor quality. Therefore, it is important to involve medical experts in the developmental stage of health-related mobile apps to improve the quality of these apps.

## Introduction

Hypertension contributes to the burden of various diseases including heart disease [1] and leads to premature mortality and morbidity [2]. Globally, 1 in 5 adults has hypertension [3], and 12.8% of total deaths worldwide are caused by high blood pressure [4]. Hypertension is a chronic disease that may result in severe complications such as stroke [5,6] and chronic kidney disease [7,8]. High blood pressure is treated in the long term pharmacologically and/or through lifestyle adjustments [9,10]. Therefore, continuous preventive care and daily management of patients is important in controlling blood pressure. Self-measured blood pressure monitoring leads to better blood pressure control [11] and enhances medication adherence [12,13]. In addition, self-measured blood pressure eliminates white coat hypertension and can be useful in the detection of masked hypertension [14].

The ownership of mobile phones is increasing rapidly and by the end of 2017, over one-third of consumers worldwide owned a mobile phone [15,16]. The health app market is growing and thousands of new health apps are published every year. In 2016, approximately 100,000 new health-related apps were published, resulting in a total of 259,000 health apps currently available in the major app stores [17]. The health app download rates were estimated to reach 3.2 billion in 2016, an increase of 7% compared to 2015 [17]. The main target areas of app developers are chronic diseases such as hypertension and diabetes [17]. A survey performed by Accenture showed that the use of health apps and wearables increased by almost 50% among consumers in 2016 compared to 2014 [18]. Furthermore, patients and physicians agree about the potential benefits of health apps and wearables [18].

Using a mobile app for self-management purposes could make it easier for patients with hypertension to have insight into and control their blood pressure. These apps may have several useful features: logbook or diary features facilitate logging of blood pressure measurements in an organized way, while reminder functions could facilitate monitoring and medication adherence. In addition, health apps may provide valuable background information for patients about the disease, its treatment, how to measure blood pressure adequately, and lifestyle management. Analysis tools (eg, graphs and trend analysis) may provide an overview of the course of blood pressure over time. Furthermore, some apps can export blood pressure readings and other user data to be sent by email. This enables patients to share their measurements with their health care providers and relatives.

Although mobile apps have the potential to be beneficial for patients with hypertension, little is known about the availability, quality, and features of mobile apps targeting blood pressure. Therefore, the aim of this study was to perform a systematic review of apps to determine the availability, functionality, and quality of mobile apps that could be used to collect readings of blood pressure for monitoring purposes.

## Methods

### App Search and Selection

We performed a systematic app search based on the standards for systematic reviews. Although we followed the standards for systematic reviews of scientific literature, these guidelines are not completely applicable to app reviews. All apps available in the Google Play store for Android and iOS App Store targeting blood pressure monitoring in which blood pressure measurements could be entered manually were potentially eligible for inclusion. On March 1, 2016, the Dutch app stores were searched using the following search terms: blood pressure, diastolic, health, heart rate, hypertension, hypotension, pressure, systolic, and their Dutch equivalents (bloeddruk, diastole, gezondheid, hartslag, hypertensie, hypotensie, and systole). Apps in languages other than English or Dutch and duplicates and irrelevant apps, such as games, were excluded. Some apps had a free version and a pro version. In cases where there was no difference in the functionality and relevant features between the two versions, only the free version was included in this app review. Two independent assessors (HJ and FRdG) selected the eligible apps based on app titles, description of the app in the app store, and screenshots provided. Discrepancies were discussed until a final decision was reached.

### Data Extraction

The selected apps were downloaded on either a Samsung Galaxy S6 (Android version 6.0.1) or an iPhone 5c (iOS version 9.3.5) for complete assessment of eligibility and characteristics. Two independent assessors (HJ and LCHL) tested each app on each platform in duplicate for a minimum of 10 minutes before performing the final assessment. Using a standardized form, the assessors recorded technical app information and app features. Recorded technical information included the name of the app, app developer, version number, platform, affiliations of the app developers, price, number of ratings of all versions in the app store, star ratings in app store, whether Web access was required, data storage location (local and/or cloud), and whether the app was free of advertisements. App features included the ability of the app to register age, gender, height, weight, time, and date of blood pressure reading, measurement site (eg, left or right arm), and measurement position (eg, sitting or standing). We also registered the presence of a reminder function, analysis functions, data export, wireless transfer of measurement data from a blood pressure monitor, and whether user data were password-protected. Based on national and international guidelines for the management of hypertension [9,19-24] and recommendations from the literature addressing the management of hypertension [25-28], we selected 6 key app features that are essential for self-management. The key features included the ability to export data, send reminders, analyze data, record time and date of blood pressure reading, record weight, and provide information/education. In addition, we searched the app descriptions in the app stores for the involvement of medical experts in the development of the app. Furthermore, we searched PubMed and Google Scholar in March 2017 to determine whether the apps were trialed or evaluated with results published in peer-reviewed journals.

### App Quality Rating

The quality of the apps was evaluated using a standard assessment protocol based on the Mobile App Rating Scale (MARS) [29], a questionnaire that measures app quality using 23 questions divided into 4 objective categories (engagement, functionality, aesthetics, and information quality) and 1 subjective category. Each question was rated on a 5-point scale (1-inadequate, 2-poor, 3-acceptable, 4-good, 5-excellent). The MARS overall score was calculated by averaging the means of the 4 objective categories. The developers of MARS recommend a training to standardize the assessors’ ratings [29], so the assessors watched the MARS training video available on YouTube [29]. Afterwards 10 randomly selected apps were used for training purposes. The assessors discussed each item of the MARS scale and reached consensus on the scores during the training. After these 10 apps, the assessors did not discuss any apps and rated them independently.

### Statistical Analysis

We calculated the scores of the MARS separately per assessor and averaged the scores at total level. The distributions of the scores were checked for normality. We measured the interrater reliability of the MARS scores using the intraclass correlation coefficient (ICC). Based on the ICC guidelines developed by Shrout and Fleiss [30], we used a 2-way mixed effects, average measures model with a consistency of agreement definition [31]. Cronbach alpha was used to assess the internal consistency reliability (ie, the extent to which all items in a scale measure the same concept) of the MARS [32].

Descriptive statistics were used to summarize and evaluate the app features. Differences in proportions were tested using chi-square tests. To determine whether specific characteristics were associated with quality scores, MARS scores of apps with and without the a priori selected characteristics were compared using independent sample *t* tests. Pearson correlation coefficients were calculated to compare the MARS overall scores with star ratings obtained from the app stores of Android and iOS. Only apps with 10 or more user ratings were included in this analysis. Statistical significance was set at *P*<.05. The data were analyzed using SPSS Statistics version 22 (IBM Corp).

## Results

### App Selection

**Figure 1:**
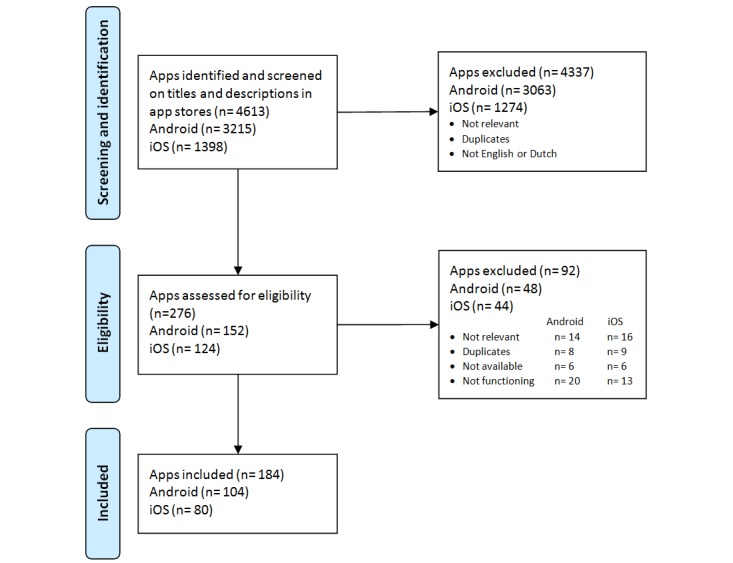
Flow diagram of the review and selection process.

A total of 4613 apps were identified using the search terms. Screening on app titles and descriptions in the app stores resulted in 276 potentially eligible apps. Further assessment, performed after downloading and testing of the selected apps, resulted in the inclusion of 184 apps, of which 104 were Android apps and 80 iOS apps. Some apps were available on both platforms so were included twice in this study. Figure 1 illustrates the selection procedure.

### App Characteristics

The app characteristics are summarized in Table 1. The majority of the apps included (77.7%, 143/184) were free of charge. Android apps were more often free compared to iOS apps (89.4% [93/104] vs 62.5% [50/80], *P*<.001). However, iOS apps were more often free of advertisements compared to Android apps (80.0% [40/50] vs 59.1% [55/93], *P*=.01). Less than half of the apps tracked personal background data, with the exception of weight tracking in Android apps. Only a small proportion of apps recorded how the blood pressure measurements were performed (measurement side, 15.2%, 28/184; position, 14.1%, 26/184). Compared to iOS apps, Android apps more often recorded measurement side (20.2% [21/104] vs 8.8% [7/80], *P*=.03) and position (19.2% [20/104] vs 7.5% [6/80], *P*=.02). Only 2 apps did not register time and date of the measurement. A reminder function was present in 28.8% (53/184) of apps, and data export was possible in 65.2% (120/184) of apps. The latter was more often available in iOS apps than in Android apps (75.0% [60/80] vs 57.7% [60/104], *P*=.02). A total of 26 apps, of which 15 were Android and 11 iOS, contained 5 predefined key features. None of the apps contained the key feature information/education.

A total of 182 apps were developed by commercial (31) or unknown (151) developers. Only 2 apps (Heartkeeper and Blood pressure monitoring for pregnancy) were developed by universities or nongovernmental organizations. Of all apps included, only 3.8% (7/184 ) stated the involvement of medical experts in the development of the app in their own app store description. None of the apps was trialed or evaluated with results published in a peer-reviewed journal.

### App Quality

The MARS overall and subscale scores were normally distributed. For iOS apps, the overall interrater reliability of the MARS was fair (ICC=0.42, 95% CI 0.21-0.59) and the Cronbach alpha was 0.59. For Android apps, the interrater reliability was slightly higher (ICC= 0.53, 95% CI 0.38-0.66) and the Cronbach alpha was 0.70. Table 2 shows the ICCs and MARS scores for the 5 categories and the MARS overall score per platform.

**Table 1:** Summary of characteristics of included apps, stratified by platform.

Characteristics		All apps (n=184), n (%)	Android (n=104), n (%)	iOS (n=80), n (%)	*P* values
**Pricing**					
	Free	143 (77.7)	92 (89.4)	50 (62.5)	<.001
	No advertisements^a^	95 (66.4)	55 (59.1)	40 (80.0)	.01
**Personal data**					
	Age	75 (40.8)	42 (40.4)	33 (41.3)	.91
	Gender	75 (40.8)	41 (39.4)	34 (42.5)	.67
	Height	71 (38.6)	44 (42.3)	27 (33.7)	.24
	Weight^b^	99 (53.8)	62 (59.6)	37 (46.2)	.07
**Blood pressure measurements**					
	Side (left or right arm)	28 (15.2)	21 (20.2)	7 (8.8)	.03
	Position (eg, sitting, lying)	26 (14.1)	20 (19.2)	6 (7.5)	.02
	Date and time^b^	182 (98.9)	103 (99.0)	79 (98.8)	.85
**Other features**					
	Reminder function^b^	53 (28.8)	30 (28.8)	23 (28.7)	.99
	Analysis tool^b^	158 (85.9)	92 (88.5)	66 (82.5)	.25
	Data export^b^	120 (65.2)	60 (57.7)	60 (75.0)	.02
	Data upload from blood pressure meter	27 (14.7)	12 (11.5)	15 (18.7)	.17
	Needs Web access to function	10 (5.4)	8 (7.7)	2 (2.5)	.12
	Password protection	43 (23.4)	20 (19.2)	23 (28.7)	.13
	Data storage (local)	181 (98.4)	102 (98.1)	79 (98.8)	.72
	Data storage (cloud)	19 (10.3)	15 (14.4)	4 (5.0)	.04

^a^Only free apps included; paid apps were presumed to have no advertisements.

^b^Key app features for self-management based on guidelines and literature.

**Table 2:** Mobile App Rating Scale scores.

MARS^a^ subscale	Android				iOS		
	Mean (95% CI)	ICC^b^ (95% CI)	Alpha^c^	Mean (95% CI)		ICC (95% CI)	Alpha
Engagement	2.28 (2.17-2.39)	.62 (.48-.72)	.76	2.26 (2.16-2.36)		.47 (.28-.63)	.64
Functionality	3.54 (3.46-3.62)	.23 (.03-.41)	.37	3.55 (3.46-3.65)		.35 (.13-.53)	.51
Aesthetics	3.06 (2.96-3.17)	.51 (.35-.64)	.68	3.16 (3.07-3.25)		.20 (.03-.40)	.33
Information	1.63 (.52-1.73)	.57 (.42-.69)	.73	1.57 (1.46-1.68)		.54 (.35-.68)	.70
Subjective quality	2.54 (2.35-2.74)	.56 (.41-.68)	.72	2.63 (2.42-2.84)		.49 (.29-.64)	.65
MARS overall score^d^	2.63 (2.55-2.71)	.53 (.38-.66)	.70	2.64 (2.56-2.71)		.42 (.21-.59)	.59

^a^MARS: Mobile App Rating Scale.

^b^ICC: intraclass correlation coefficient.

^c^Alpha: Cronbach alpha.

^d^Average of 4 objective subscales.

**Table 3:** Quality scores comparison of apps with and without a specific characteristic.

Characteristics		Present				Not present				Difference	
			n (%)		Mean (SD)		n (%)		Mean (SD)		Mean (95% CI)
**Pricing**											
	Free		143 (77.7)		2.66 (.38)		41 (22.3)		2.55 (.33)		.11 (–.24 to .02)
	No advertisements^a^		95 (66.4)		2.67 (.38)		48 (33.6)		2.64 (.37)		.03 (.10 to .16)
**Personal data**											
	Age		75(40.8)		2.78 (.39)		109 (59.2)		2.53 (.32)		.25 (.15 to .35)
	Gender		75 (40.8)		2.76 (.36)		109 (59.2)		2.54 (.35)		.22 (.11 to .32)
	Height		71 (38.6)		2.74 (.35)		113 (61.4)		2.56 (.37)		.18 (.07 to .28)
	Weight^b^		99 (53.8)		2.74 (.37)		85 (46.2)		2.50 (.33)		.24 (.14 to .34)
**Blood pressure measurements**											
	Side (left or right arm)		28 (15.2)		2.83 (.40)		156 (84.8)		2.60 (.35)		.23 (.09 to .38)
	Position (eg, sitting, lying)		26 (14.1)		2.87 (.42)		158 (85.9)		2.60 (.35)		.27 (.12 to .42)
	Date and time^b^		182 (98.9)		2.64 (.36)		2 (1.1)		2.04 (.61)		.60 (.08 to 1.01)
**Other features**											
	Reminder function^b^		53 (28.8)		2.82 (.36)		131 (71.2)		2.56 (.35)		.26 (.15 to .38)
	Analysis tool^b^		158 (85.9)		2.67 (.37)		26 (14.1)		2.40 (.26)		.27 (.12 to .42)
	Data export^b^		120 (65.2)		2.71 (.39)		64 (34.8)		2.48 (.28)		.23 (.13 to .35)
	Data upload from blood pressure meter		27 (14.7)		2.94 (.35)		157 (85.3)		2.58 (.35)		.36 (.22 to. 51)
	Needs Web access to function		10 (5.4)		2.88 (.40)		174 (94.6)		2.62 (.36)		.26 (.03 to .50)
	Password protection		43 (23.4)		2.78 (.38)		141 (76.6)		2.59 (.38)		.19 (.07 to .32)
	Data storage (local)		181 (98.4)		2.62 (.36)		3 (1.6)		3.10 (.52)		–.48 (–.89 to –.05)
	Data storage (cloud)		19 (10.3)		3.04 (.46)		165 (89.7)		2.58 (.33)		.46 (.29 to .62)

^a^Only free apps included; paid apps were presumed to have no advertisements.

^b^Key app features for self-management based on guidelines and literature.

On a scale from 1 to 5, the mean MARS score for the 4 objective categories was 2.6 for both platforms. The MARS scores for the separate categories were also very similar for Android and iOS. Subjective quality scored 2.5 and 2.6 for Android and iOS, respectively. Of the 5 categories, functionality received the highest score (Android 3.5, iOS 3.6) and information the lowest (Android 1.6, iOS 1.6). The complete list is available in Multimedia Appendix 1.

Among the 80 iOS apps, 7 had 10 or more user ratings in the app store, but we did not observe a correlation between the star ratings and the MARS overall score (*r*=0.29; *P*=.53). For Android, 78 apps received ≥10 user ratings; these were not correlated with the MARS scores either (*r*=0.17; *P*=.15). Table 3 shows the quality scores comparison for apps with and without each characteristic. Incorporation of all characteristics resulted in higher quality scores except for pricing, in-app advertisements, and local data storage.

### App Top 5

The 5 apps with the highest MARS overall scores per platform are listed in Multimedia Appendix 2 together with their characteristics. On Android, Bloeddruk (developer: Klimaszewski Szymon) and Beurer HealthManager (developer: Beurer) were the best-scoring apps, with MARS overall scores of 4.1 and 3.7, respectively. AMICOMED BP (developer: AMICOMED) was the best-scoring app on the iOS platform with an overall score of 3.6. All apps in the top 5 for each platform were free.

## Discussion

### Principal Findings

In this study, we observed a lower MARS overall score compared to other reviews focusing on apps for other self-management aspects [33-35]. However, comparable to our study, functionality was previously reported as the objective category with the highest MARS score [33,35]. Our results also showed that some of the app features have a large influence on the overall quality score. The app features with the most positive influence on the app quality score are the ability of using the cloud for data storage, wireless data upload from blood pressure meters, ability to export data, ability to analyze data, ability to send reminders, and ability to record personal data, such as age and weight. More than half of the apps can export data and approximately 15% of the apps were able to upload data from blood pressure meters. If present, the latter feature makes it easier and more convenient to measure and record blood pressure.

Only approximately a quarter of the apps in our study had a reminder function, but reminder features can be very important in facilitating adherence [36,37]. The authors of a previous study on hypertension apps reported similar results [38]. All of the selected key features except information/education resulted in a large positive influence on the app overall quality score. The information/education feature was often absent or of poor quality in the apps included in this study. Only 2 apps were developed by a university or nongovernmental organizations and none of the apps was evaluated with results published in the literature. This, combined with the low scores for information on the MARS scale, suggests the lack of involvement from medical experts in the process of app development, which was also reported in previous studies [39-42]. The high MARS scores for functionality combined with the low MARS scores for information suggests that most apps function well but lack important information. This lack of information may result into incorrect use of the app (eg, incorrect interpretation of blood pressure readings, resulting in potential nonadherence to therapy) by users who are not sufficiently literate in digital health. Therefore, health apps should be validated before use. We found suboptimal ICCs and Cronbach alphas for the functionality subscale in Android and the aesthetics subscale in iOS. An explanation for the suboptimal ICCs and Cronbach alphas may be a lower agreement between assessors for the functionality and aesthetics subscales due to discrepancies on subjective criteria between the assessors, which arose after the training used to standardize the assessors’ ratings. Another explanation may be that the MARS is not a perfect instrument to assess these features. The developers of MARS also reported a lower ICC for the functionality subscale [29].

There were no differences in quality scores between paid and free apps, which has also been reported previously [35]. Notably, the top 5 apps, identified by the highest MARS overall scores, were all free of charge. However, developers may earn money with their apps by selling the data shared by users and/or by promoting other products that can be combined with their apps, such as blood pressure meters. PatientsLikeMe is a well-known example. This platform is free of charge and very useful, but user data is sold [43]. That may explain why these apps are offered free of charge in the app stores by the app developers. We did not observe a correlation between the star ratings and the MARS overall scores. However, it is difficult to assess reliability of star ratings in app stores, since the criteria and qualifications of assessors are not always clear. For example, reviewers may leave ratings that do not reflect their true opinions or only selected users leave a rating (selection bias). The authors of a recent study on app store user ratings and reviews of a blood pressure app (Instant Blood Pressure) reported that these types of ratings were unreliable [44].

In this study, we identified a large number of apps ineligible for self-management and many apps of poor quality. These apps may potentially be harmful to users. Apps providing patients with erroneous information or apps that do not do what they are supposed to do are examples of such harmful apps. At the American Medical Association interim meeting in 2016, Executive Vice President James Madara mentioned a blood pressure app that failed at high rates in detecting elevated blood pressure and yet was one of the most frequently downloaded health apps for 2 years [45]. It is important to separate good apps from the harmful ones and to stimulate the development of high quality apps. Performing systematic app reviews and/or developing guidelines for health app developers could reduce the development of poor-quality health apps. It is important to regulate the development of health apps internationally, because apps are available in multiple national app stores. Therefore, setting up an international institute to regulate the development of health apps or certifying health apps may be necessary. The Health On the Net Foundation (HON) is a good example of such an institute. HON assesses the quality of health information online and provides certification to websites with reliable health information [46].

### Strengths and Limitations

A major strength of this study is that we searched the 2 main app stores systematically using 15 search terms in English and Dutch and included both paid and free apps. This resulted in a large number of apps that were first screened on titles and descriptions in the app stores. All apps identified through this process were assessed by 2 independent reviewers. In addition, we assessed the quality of the apps objectively using MARS [29], which has previously been used to evaluate app quality in several app reviews [35,47,48].

This review was limited to Dutch app stores, and we included apps in English or Dutch only. It is possible that other national app stores may contain a larger, smaller, or different assortment of apps. Although it is not feasible to search all national app stores from a single country, most apps are released worldwide and are not country-specific. Furthermore, we limited our search to the major app platforms Android and iOS. These platforms, however, accounted for approximately 98% of the mobile phone market share in 2015 [49]. In addition, we excluded apps that need a prescription by a health care provider or permission for use from the developer. Therefore, we may have missed potentially eligible apps, but these are not generally available to the target population. Another limitation was the compatibility of apps. As a large variety of mobile phones with several software versions are available in the markets, some apps may not have been compatible with the devices used in this study. However, it is not feasible to assess all apps using a large spectrum of mobile phones. We used the most recent software versions to ensure the maximum stability and safety.

### Perspectives

Mobile apps may be a useful tool for self-management for patients with hypertension. In addition, mobile apps could be used to provide information to patients and increase awareness about blood pressure–related health issues among patients. Also, many mobile apps can export blood pressure data, which could be used by health care providers to make more informed decisions regarding treatment [50,51]. Furthermore, patients will be more involved in their own treatment through the use of high-quality, dedicated mobile apps. Therefore, health care providers should stimulate the use of mobile apps by patients with hypertension. In that case, however, they have to be sure that the apps used by their patients do not contain any misleading or harmful information. App reviews could be a suitable instrument to separate the useful apps from the harmful ones. Nevertheless, a practical guideline for app reviews is not available. Therefore, it is crucial to develop an international guideline for performing app reviews. This study provides a list of the top 5 useable apps targeting blood pressure monitoring available on the 2 major mobile phone platforms. Health care providers and patients with hypertension can use the results presented in this study to identify a suitable high-quality app targeting blood pressure monitoring, provided that blood pressure measurements are valid.

### Conclusion

In this review, we identified only a few apps with sufficient quality for blood pressure self-management purposes. The use of these sufficient quality apps should be stimulated to improve patient care. This study provides an overview of the best apps currently available in the app stores and important key features for self-management that can be used by health care providers and patients with hypertension to identify a suitable app targeting blood pressure monitoring. However, the majority of the apps targeting blood pressure monitoring were of poor quality, and the accuracy of the blood pressure measurements registered in the apps was not assessed. It is important to involve medical experts in the developmental stage of health-related mobile apps to improve the quality of these apps.

## Appendix

Multimedia Appendix 1 The complete list of the reviewed apps and Mobile App Rating Scale overall scores.

Multimedia Appendix 2 Top five hypertension apps on the iOS and Android platforms.

## Abbreviations

HON: Health on the Net
ICC: intraclass correlation coefficient
MARS: Mobile App Rating Scale
