# Supplementary material for: Mobile Apps for Blood Pressure Monitoring: Systematic Search in App Stores and Content Analysis
Source: JMIR Mhealth Uhealth. 2018 Nov 14;6(11):e187. doi: 10.2196/mhealth.9888 (PMC6262205; doi:10.2196/mhealth.9888)
Supplement: Multimedia Appendix 1 [file mhealth_v6i11e187_app1.pdf]

Platform	App name	App developer	MARS overall score
Android	Bewaken Mijn BP	APG Solutions, LLC	2.0
Android	Bloeddruk Inloggen	Aronegy	2.1
Android	Bloeddruk Log Dagboek	PureStyle360	2.7
Android	Blood Pressure	Bemo Apps	2.4
Android	Blood Pressure	Freshware	2.8
Android	Blood Pressure	Gennaro Guarino	2.4
Android	Blood Pressure - Heartcare	Aman Shivhare	3.1
Android	Blood Pressure (BP) Report	TriStarApps	2.5
Android	Blood Pressure (BP) Watch	NumbersMatter2Me	3.2
Android	Blood Pressure (SmartBP)	Evolve Medical Systems	3.1
Android	Blood Pressure Dairy	Ser	2.2
Android	Blood Pressure Dairy	Umbrella Corp Android	2.6
Android	Blood Pressure Diary	FRUCT	2.9
Android	Blood Pressure Down	Calories LLC	2.4
Android	Blood Pressure Journal	3qubits	3.3
Android	Blood Pressure Log	jucdejeb	2.9
Android	Blood Pressure Log - MyDiary	Dr Tomasz Jan Zlamaniec	3.3
Android	Blood Pressure Log - Trial	Sgadan Creek Software	2.1
Android	Blood Pressure Logger	Daffodil Technologies (I) Pvt Ltd	2.7
Android	Blood Pressure Logger	Time2Relax	2.3
Android	Blood Pressure Logger (Health)	Pixelogic Apps	2.7
Android	Blood Pressure manager	(?_-*)???	2.0
Android	Blood Pressure Meds SciTracker	Justin Taylor Dev	3.1
Android	Blood pressure memo	Mirko Martinović	2.4
Android	Blood Pressure Monitor Diary	Boost Developers	2.3
Android	Blood Pressure Monitoring	Juan B and Juan H Android Development	2.3
Android	Blood Pressure QuickLog.me	Genehome	2.5
Android	Blood Pressure Record free	Kmcpesh apps	2.5
Android	Blood Pressure Records	Allan Mosiro	2.2
Android	Blood Pressure Tab	Catimos	2.5
Android	Blood Pressure Tracker	aadhk	3.1
Android	Blood Pressure Tracker	California Healthcom Group	2.3
Android	Blood Pressure Tracker	Refael Sheinker	2.5
Android	Blood Pressure Tracker	Sparkle Solutions	1.7
Android	BLOOD PRESSURE TRACKER SYSTEM	Cyblance Technologies Private Limited	2.6
Android	Blood Pressure(BP) Diary	Cufit Inc.	3.0
Android	BloodPressure Vue	vue	3.0
Android	BloodPressureDB	Horst Klier	2.6
Android	Bluetooth Blood Pressure	IdeaSynthesis LLC	2.3
Android	BP Log Tracker	Abhisoft Tech	2.5
Android	BP Tracker-Symptoms & Solution	Droid Studio India	2.7
Android	BPMonitor	NowPos	2.5
Android	C.A.P.D. Helper	eeighon	2.7
Android	Cardio Journal blood pressure	MDHELPER	3.3
Android	Cardiograph diary pro	Double A Developers	2.2

Android	DailyChek Cardio Health Free	hlmt rd	2.9
Android	dbees.com Diabetes Management	Freshware	2.3
Android	Diabetes & Blood Pressure Log	Cooey Technologies	2.4
Android	Diabetes BP Health Tracker App	Justin Taylor Dev	2.6
Android	Digital Cardiograph diary pro	Double A Developers	2.4
Android	Dr. Mohan's Diabetes App	Jana Care	2.7
Android	Easy Blood Pressure Diary	SILECI Apps	2.9
Android	EHR / EMR Health records	AvvaStyle	2.6
Android	eHypertension Pro free	Physiosensing, Inc	2
Android	EZ Health Tracker	Ed Avoundjian	2.4
Android	Fitga Fitness Tracker	KyaliApps	2.2
Android	GO - Gezondheid & Geschiktheid	DROID INFINITY	2.8
Android	Habits: Diabetes Coach	Jana Care	2.7
Android	Health Assistant	WSMRSOFT	2.9
Android	Health Diary	ORIN	2.6
Android	Health Diary	PNN Soft	2.8
Android	Health Manager "Healthiern"	Kawashore	2.2
Android	Health Master Free	GreenLife Apps	2.4
Android	Health Mate	Withings	2.7
Android	Health Measure Graph	Soft Industry	2.7
Android	Health Report Daily	Elapse Technologies	2.5
Android	Health Tracker & Manager App	XLabz Technologies Pvt Ltd	3.2
Android	Health Tracking - Pow Health	Pow Health	2.6
Android	Health-PIE Digital Nurse	mTatva - Health App & Fitness App	2.8
Android	Health-Tracker	Lior Ben Oved	2.8
Android	HealthWatch 360	GB HealthWatch	3.1
Android	Heart Manager	BlueBird Apps	2.8
Android	Heartkeeper	Grupo de Telemedicina y eSalud de la UVa	2.7
Android	Heartservice	Kenkou	2.6
Android	iBP Blood Pressure	Leading Edge Apps LLC	3.0
Android	Logboek bloeddruk	bplog.it	2.7
Android	Medilio	Casperise	2.8
Android	Medische geschiedenis dossiers	xHealth	2.8
Android	MedM Blood Pressure	MedM Inc	3.3
Android	MedM Health	MedM Inc	2.6
Android	Mijn Bloeddruk	3 ACORN Technologies, LLC	1.6
Android	My Blood Pressure	Konviere Corporation	2.4
Android	My Blood Pressure	Mengtao Ye	2.7
Android	My Blood Pressure & Heart Rate	Webpatient	2.3
Android	My Health Tracker	Walter Gross	2.5
Android	myBloodPressure Free	Arbitrary Software LLC	2.7
Android	Pocket Health Tracker	wide apps	2.6
Android	Pressure log	X64h	2.2
Android	Pressure Monitor	And Developer	2.3
Android	PressureLog	apps4use	2.5
Android	S Health	Samsung Electronics Co., Ltd.	3.4

Android	Sanitas HealthCoach	Hans Dinslage GmbH	3.2
Android	Simple Blood Pressure Tracking	TheTracker	1.8
Android	TactioGezondheid	Tactio Health Group	3.1
Android	AppVita	AViTA Corp.	2.3
Android	Beurer HealthManager	Beurer GmbH	3.7
Android	Bloed dagboeken	EONSOFT	2.5
Android	Bloeddruk	Klimaszewski Szymon	4.1
Android	Blood Pressure Diary	LABC FACTORY	2.3
Android	Blood Pressure Diary	Mobispin	2.3
Android	Blood Pressure Diary	OrangeOlive	2.6
Android	Blood pressure diary - free!	Androcalc	2.6
Android	blood pressure healthy	tdtomlou	2.4
Android	ContinuousCare Health App	NeedStreet	2.9
iOS	AMICOMED BP	AMICOMED	3.6
iOS	Braun Healthy Heart	Kaz USA, Inc.	3.5
iOS	Blood Pressure	Evolve Medical Systems, LLC	3.3
iOS	Beurer HealthManager	Beurer GmbH	3.3
iOS	Health Mate - Steps tracker & Life coach door Withings	Withings	3.2
iOS	AGR Blood Pressure Log	angel garcia rubio	3.1
iOS	HeartStar Blood Pressure Monitor	Little Green Software	3.1
iOS	iBP Blood Pressure	Leading Edge Apps LLC	3.1
iOS	Health Tracker & Manager for iPhone - Personal Healthbook App for Tracking Blood Pressure BP, Glucos	XLabz Technologies Pvt. Ltd.	3.0
iOS	Blood Pressure Monitor - Family Lite	Taconic System LLC	3.0
iOS	Blood Pressure Logger	suprabhat chouksey	3.0
iOS	Bloeddrukmeter FREE	Dzmitry Permiakou	2.9
iOS	BPMon - Blood Pressure Monitor	Anatoly Butko	2.9
iOS	Bloody Pressure	Piotr Sochalewski	2.9
iOS	Sanitas HealthCoach	Hans Dinslage GmbH	2.9
iOS	BodyGauge	Martin Edelbroek	2.9
iOS	Best Blood Pressure Monitor	Andrei Mitrohin	2.9
iOS	Blood Pressure Monitor - Cloud Edition	Thomas Kress	2.9
iOS	MedM Blood Pressure	SwissMed Mobile AG	2.9
iOS	Cardio+	Microlife Corp.	2.8
iOS	Blood Pressure Tracker QuickLog.me	Genehome	2.8
iOS	Daily Carb - Carbohydrate, Glucose, Medication, Blood Pressure and Exercise Tracker	Maxwell Software	2.8
iOS	Bloeddruk Assistent - Bloeddrukmetingen registreren en bijhouden	Josef Moser	2.8
iOS	BP Wiz – Blood Pressure Log and Medication Tracker	LINKLINKS LTD	2.8

iOS	Kang BP	kangkang	2.8
iOS	Chart My BP - Blood Pressure Tracker	APPSTRUCTURE LLC	2.8
iOS	Bloodnote - Blood pressure control	Matt Ludzen and Peter Bajtala	2.8
iOS	Blood Pressure Companion	Maxwell Software	2.7
iOS	Body Logger	Jerry Juang	2.7
iOS	Blood Pressure Track	Feeltracker Ltd	2.7
iOS	Control Tension	Les Laboratoires Servier	2.7
iOS	Tactio Gezondheid: Mijn Aangesloten Gezondheid Logboek	Tactio Health Group Inc.	2.7
iOS	HoMedics	HoMedics	2.7
iOS	Diabetes Pedometer with Glucose & Food Diary, Weight Tracker, Blood Pressure Log and Medication Remi	Pacer Health, Inc	2.7
iOS	Glucose Buddy Pro : Diabetes Managing Logbook w/ Blood Pressure & Weight Tracking	Azumio Inc.	2.7
iOS	Blood Pressure Logger (Quick & Simple Series)	Wonho Park	2.7
iOS	Bloeddruk Bijhouden	Japps	2.6
iOS	myBloodPressure	Arbitrary Software, LLC.	2.6
iOS	bp Trax® – Blood Pressure Treatment Tracker, Lifestyle, Resting, BMI, MAP, Pulse Rate	iMobLife Inc.	2.6
iOS	Blood Pressure Tracker - Pro Version	iHealth Ventures LLC.	2.6
iOS	iHeart - Pulse Reader	Anatoly Butko	2.6
iOS	Blood Pressure & Pulse Diary	Jan-Hendrik Damerau	2.6
iOS	myDiabetes	Urban E-Learning Pty Ltd	2.6
iOS	Blood Pressure Logs	Appicasso	2.6
iOS	iCardio GPS, Run, Walk, Bike and Exercise Heart Rate Calorie Monitor and Health Fitbit Tracker	iTMP Technology, INc.	2.6
iOS	myVitali Gezondheidscoach - Vergelijk Fitness en Activiteit	myVitali AG	2.6
iOS	Easy BP	Moonstone Apps	2.6
iOS	BloodRate	Ken Madsen	2.56
iOS	Blood Pressure	Bartholomaeus Maciag	2.5
iOS	Laborom - diabetes, bloed druk logboek	xHealth	2.5
iOS	Reframe Health Center - For patients with chronic diseases	Reframe	2.5
iOS	BP Buddy - 80% OFF SALE - Blood Pressure helper	Azumio Inc.	2.5
iOS	120 over 80	Jeffrey Kempster	2.5
iOS	Your Blood Pressure	WWW Machealth Pty Ltd	2.5
iOS	BP Recorder	Cappable Limited	2.5
iOS	MyDASHDiet	Fresh Object Software	2.5

iOS	Blood Pressure Monitoring for Pregnancy	Incentivated	2.5
iOS	My Blood Pressure Readings	Puig Labs	2.5
iOS	Diabetes Kit Blood Glucose Logbook - Burn Calories & Lower Sugar Levels with Advanced Pedometer Trac	Diabetes Labs, LLC	2.4
iOS	Quick BP	Evocomputing Inc.	2.4
iOS	Easy Blood Pressure	Seaside Apps	2.4
iOS	Blood Pressure Monitor	Afanche Technologies, Inc.	2.4
iOS	GenieMD	GenieMD, LLC	2.4
iOS	Gezondheid log keeper	A1Brains Infotech	2.4
iOS	Blood Pressure Made Easy	Chad Dunlap	2.4
iOS	Bloed dagboek (Diabetes Management)	Seong Eon Kim	2.4
iOS	Blood Pressure Passport free	ecoTouchMedia.com	2.4
iOS	Blood Pressure Down	Calories, LLC	2.3
iOS	Easy Blood Pressure Diary	SILECI	2.3
iOS	mHypertension	CRT Technology	2.3
iOS	Goal Blood Pressure	benjamin hysell	2.3
iOS	How Am I Doing Today	Jiajie Li	2.3
iOS	Blood Pressure+Pulse Grapher Lite	michael heinz	2.3
iOS	Monitor Mijn BP door APG Solutions LLC voor iPhone en iPad	APG Solutions, LLC	2.2
iOS	Hypertension & Diabetes	eclaireMD	2.2
iOS	Live Heart - Blood Pressure measurements with related symptoms	Gabriel Gamil	2.1
iOS	Blood Pressure Diary 2	cellHigh	2.1
iOS	120/70: Blood Pressure Logging Helper	SERGIY SKOBLIKOV	2.1
iOS	Simple Blood Pressure Recorder	Stack3	2.0
iOS	Blood Pressure Register	Manu Gupta	1.8
