# Supplementary material for: Mobile Apps for Blood Pressure Monitoring: Systematic Search in App Stores and Content Analysis
Source: JMIR Mhealth Uhealth. 2018 Nov 14;6(11):e187. doi: 10.2196/mhealth.9888 (PMC6262205; doi:10.2196/mhealth.9888)
Supplement: Multimedia Appendix 2 [file mhealth_v6i11e187_app2.pdf]

Multimedia Appendix 2. Top five hypertension apps on the iOS and Android platforms.

App characteristic	Android					iOS				
	Bloeddruk (Klimaszewski Szymon)	Beurer HealthManager (Beurer GmbH)	S Health (Samsung Electronics Co)	Cardio Journal blood pressure (MDHELPER)	MedM Blood Pressure (MedM Inc)	AMICOMED BP (AMICOMED)	Braun Healthy Heart (Kaz USA, Inc)	Blood Pressure (Evolve Medical Systems)	Beurer HealthManager (Beurer GmbH)	Health Mate (Withings)
**MARS overall score**	4.1	3.7	3.4	3.3	3.3	3.6	3.5	3.3	3.3	3.2
**Pricing**										
Price	Free	Free	Free	Free	Free	Free	Free	Free	Free	Free
Ads free		X	X	X	X	X	X		X	X
**Personal data**										
Age	X	X		X	X	X	X	X	X	X
Gender		X		X	X	X	X	X	X	X
Height		X	X	X				X	X	X
Weight[a]	X	X	X	X			X	X	X	X
**BP measurements**										
Side (left or right arm)	X				X			X		
Position (e.g., sitting, lying)	X				X			X		
Date and time[a]	X	X	X	X	X	X	X	X	X	X
**Other features**										
Reminder[a]	X			X		X	X	X		X
Analysis tool[a]	X	X	X	X	X	X	X	X	X	X
Data export[a]	X	X	X	X	X	X	X	X	X	X
Data upload from BP-meter		X			X	X		X	X	X
Password protection		X	X		X	X			X	X

[a] Key app features for self-management based on guidelines and literature.
